# Activation of AMPA Receptors in the Suprachiasmatic Nucleus Phase-Shifts the Mouse Circadian Clock *In Vivo* and *In Vitro*

**DOI:** 10.1371/journal.pone.0010951

**Published:** 2010-06-03

**Authors:** Yasutaka Mizoro, Yoshiaki Yamaguchi, Rena Kitazawa, Hiroyuki Yamada, Masahiro Matsuo, Jean-Michel Fustin, Masao Doi, Hitoshi Okamura

**Affiliations:** 1 Department of Systems Biology, Kyoto University Graduate School of Pharmaceutical Sciences, Kyoto, Japan; 2 Division of Molecular Brain Science, Kobe University Graduate School of Medicine, Kobe, Japan; Vanderbilt University, United States of America

## Abstract

The glutamatergic neurotransmission in the suprachiasmatic nucleus (SCN) plays a central role in the entrainment of the circadian rhythms to environmental light-dark cycles. Although the glutamatergic effect operating via NMDAR (*N*-methyl D-aspartate receptor) is well elucidated, much less is known about a role of AMPAR (α-amino-3-hydroxy-5-methylisoxazole-4-propionic acid receptor) in circadian entrainment. Here we show that, in the mouse SCN, *GluR2* and *GluR4* AMPAR subtypes are abundantly expressed in the retinorecipient area. *In vivo* microinjection of AMPA in the SCN during the early subjective night phase-delays the behavioral rhythm. In the organotypic SCN slice culture, AMPA application induces phase-dependent phase-shifts of core-clock gene transcription rhythms. These data demonstrate that activation of AMPAR is capable of phase-shifting the circadian clock both *in vivo* and *in vitro*, and are consistent with the hypothesis that activation of AMPA receptors is a critical step in the transmission of photic information to the SCN.

## Introduction

Circadian oscillations within the neuronal networks of the suprachiasmatic nucleus (SCN) and their entrainment to environmental time-cues are key features of the circadian system [1], [2]. Autonomous oscillations, generated by clock genes interlocked in transcription/translation feedback loops in the SCN [3], [4], are synchronized to environmental light-dark cycles. The retinohypothalamic tract conveys photic information to the SCN [5], [6]. Glutamate is thought to be the main transmitter in this pathway [7], [8], since optic nerve stimulation *in vitro* increases the release of ^3^H-glutamate from the retinohypothalamic terminals in the SCN [9]. Supporting this view, the two principal ionotropic glutamate receptors, α-amino-3-hydroxy-5-methylisoxazole-4-propionic acid receptor (AMPAR) and *N*-methyl D-aspartate receptor (NMDAR) are known to be localized in the SCN [10].

The phase-shifting effect of NMDAR activation has been thoroughly investigated. NMDA microinjection in the hamster SCN was shown to produce light-like phase shifts of circadian locomotor activity rhythms [11]. When applied to SCN slices *in vitro*, NMDA induced phase shifts in the rhythms of neuronal firing rate [12], [13]. The phase-shifting effect of NMDA on core-clock oscillatory rhythms was also observed in real-time monitoring system using organotypic SCN slice cultures obtained from transgenic mice expressing luciferase under the control of the core clock gene *Period1* promoter (*Per1-luc*) [14]. Together with the findings that the pretreatment of the SCN with a NMDAR antagonist prevents light-induced phase shifts in mice and hamsters [15], [16], it is believed that photic information processing relies on NMDAR-mediated neurotransmission in the entrainment of behavioral rhythms.

In contrast, the effect of AMPAR signaling on photic entrainment is still obscure. Since *in vivo* application of an AMPAR antagonist prevents light-induced phase shifts of the locomotor activity rhythms [16], AMPAR signaling is likely to contribute to photic entrainment. Yet, AMPAR signaling appears to be only partially involved in NMDAR mediated signals, since an AMPAR antagonist had only a partial inhibitory effect on NMDA-induced phase shift [11]. This is in line with the general view of glutamatergic transmission [17], which begins with a fast response generated by AMPAR [18], and the resultant membrane depolarization then leads uncoupling of magnesium block of NMDAR channels to allow calcium entry into the neurons [19], [20], suggesting the role of AMPAR activation is just a prerequisite for NMDAR activation.

Does AMPAR signaling *per se* have the ability to induce phase-shifts? In the SCN slices *in vitro*, Shibata and coworkers demonstrated that AMPA application induced phase-shifts of neuronal firing rhythms similar to NMDA [13]. Moreover, it was recently shown that AMPA application increased the calcium concentration in the SCN slices [21]. These studies prompted us to re-evaluate the effects of AMPA both on the regulation of phase-shifts of behavioral rhythms and on clock gene expression. Here we report: 1) localizations of cells expressing each subtype of AMPAR in the mouse SCN, 2) the effect of AMPA-microinjection in the SCN on locomotor activity rhythms, and 3) the effect of AMPA on core clock gene expression rhythm using the real-time monitoring system of *Per1-luc* SCN slice cultures. These results provide evidence that activation of AMPAR *per se* is capable of phase-shifting the circadian clock both *in vivo* and *in vitro*, and highlight the contributions of AMPA receptor signaling which might have been underestimated behind NMDA receptor signaling.

## Results

### Expression of AMPA receptors in the mouse SCN

**Figure 1:**
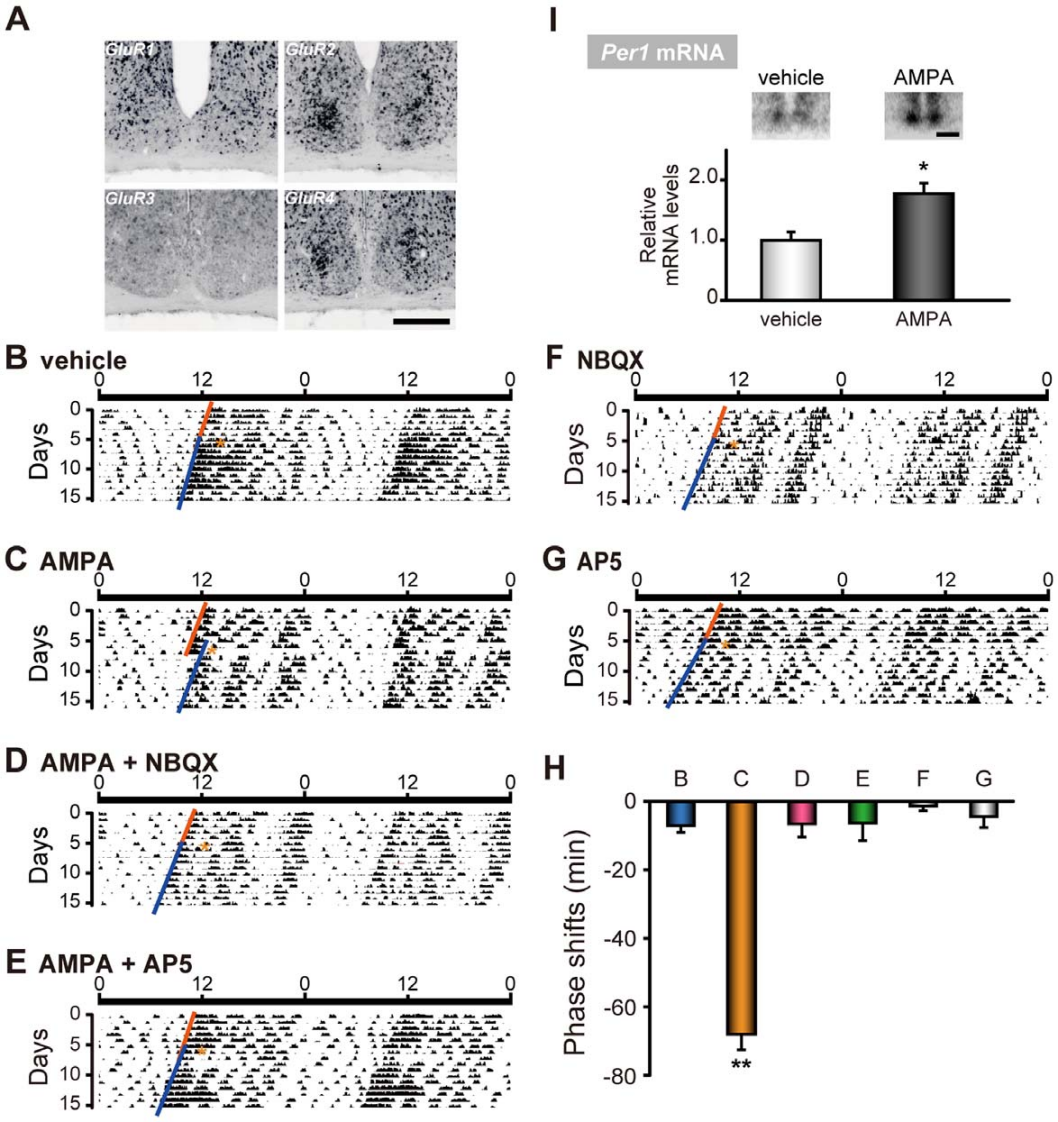
AMPA microinjection at CT14 induced phase delays and *Per1* expressions *in vivo*. (**A**) Topographic analysis of AMPA receptor mRNA expressions (*GluR1-4*) in the mouse SCN by *in situ* hybridization using digoxigenin-labeled riboprobes. Scale bar, 200 µm. (**B–G**) Representative double-plotted actograms of circadian locomotor activity rhythms in mice injected with either (**B**) vehicle, (**C**) AMPA, (**D**) AMPA + NBQX, (**E**) AMPA + AP5, (**F**) NBQX or (**G**) AP5. Mice were maintained in constant darkness and microinjections were given at CT14 (marked by asterisks) under dim red light illumination. The magnitude of the phase delays was calculated by comparing eye-fitted lines drawn according to the onset of the locomotor activity before and after the microinjection. (**H**) Summary of phase delays (Mean ± SEM) induced by microinjection of drugs at CT14. Minus values mean phase delays. Numbers at the bars denote sample sizes for each condition. ** *p*<0.01 (one-way ANOVA, followed by Scheffe's multiple comparisons). (**I**) Acute induction of *Per1* mRNA (Mean ± SEM) induced by AMPA or vehicle microinjection, detected by *in situ* hybridization using [^33^P]-labeled riboprobes. The average value of vehicle microinjection was set to 1. * *p*<0.05 (Student's *t*-test). Inset panels show representative autoradiograph images of *Per1* mRNA expression induced by vehicle (left) or AMPA (right) microinjection at CT14. Scale bar, 500 µm.

First, we examined the cellular expressions of each AMPAR subunit in the mouse SCN using *in situ* hybridization with digoxigenin-labeled riboprobes. *GluR2* and *GluR4* mRNA were highly expressed in densely distributed neurons of the middle to ventrolateral region of the SCN **(** **Figure 1A** **)**. *GluR1* was expressed moderately in the dorsal and very sparsely in the ventral SCN, and *GluR3* was not detected at all. The expression pattern of *GluR2* and *GluR4* AMPARs suggest a role for these receptor subtypes in entrainment, since ventrolateral neurons receive inputs from glutamatergic retinohypothalamic nerve endings [22], [23].

### AMPA-induced behavioral phase shifts

In order to examine the role of AMPAR signaling in the entrainment of mouse circadian locomotor activity rhythms, we directly microinjected AMPA in the mouse SCN via a pre-implanted cannula. It is known that light-induced phase-shifts are maximum in the early night in this strain of animals [24]. Therefore, we administered AMPA at circadian time (CT) 14 (2 hours after the beginning of the subjective night at CT12, defined as the time of locomotor activity onset). *In vivo* microinjection of AMPA in the SCN resulted in a delay of circadian locomotor activity rhythms when delivered at CT14 (AMPA, −67.8±4.6 min, n = 8; vehicle, −7.4±1.6, n = 3; negative and positive values represent phase delays and phase advances, respectively) **(** **Figure 1B, C** **)**. The AMPA-induced phase delays were completely inhibited by coadministration of an AMPA receptor antagonist, 2,3-dioxo-6-nitro-1,2,3,4-tetrahydrobenzo[f]quinoxaline -7-sulfonamide (NBQX) (AMPA + NBQX, −6.6±3.8 min, n = 3) **(** **Figure 1D** **)**. Microinjection of NBQX alone did not significantly induce phase shift (NBQX, −1.4±1.4 min, n = 3) **(** **Figure 1F** **)**.

Since the magnitude and directions of phase-shifts are known to vary depending on the time of light stimulation [24], we also microinjected AMPA at different circadian times. AMPA microinjection did not induce phase-shifts both at CT6 (AMPA, −1.5±0.5 min, n = 4; vehicle +1.4±4.1 min, n = 3) and at CT22 (AMPA, +5.0±2.9 min, n = 3; vehicle, −2.4±1.4 min, n = 3) **(Figure S1)**. Together, these findings indicate that the action of AMPAR signaling is phase-dependent, with a pronounced phase-delaying effect during the early night when light stimulation also induces phase delays.

To examine the involvement of NMDAR in the AMPAR mediated phase-delays at CT14, we co-administered a NMDAR antagonist, (2R)-2-amino-5-phosphono-pentanoic acid (AP5), simultaneously with AMPA. AMPA-induced phase-delays were completely inhibited by coadministration of AP5 (AMPA + AP5, −6.3±5.2 min, n = 4) **(** **Figure 1E** **)**. Microinjection of AP5 alone did not produce significant phase shifts (AP5, −5.0±2.4 min, n = 5) **(** **Figure 1G** **)**. These findings suggest that AMPAR activation *per se* subsequently activates NMDAR, which is also necessary for the AMPA-induced phase-shift.

### AMPA rapidly induces clock gene in the SCN

The key role of *Per1* in entrainment was speculated from the evidences that the application of *Per1* antisense oligonucleotides blocked the light-induced phase shifts of the behavioral rhythms [25], [26]. Indeed, the rapid induction of *Per1* was observed after a phase-shifting light exposure [27] or NMDA microinjection in the SCN [28]. Here we examined the expression of this gene after AMPA microinjection to the SCN at CT14, and found that AMPA rapidly induced higher levels of *Per1* mRNA in the SCN compared to vehicle-injected SCN (vehicle, 1.00±0.14, n = 3; AMPA, 1.78±0.14, n = 3) **(** **Figure 1I** **)**.

### AMPA-induced phase-dependent phase-shift of core clock transcription rhythms in SCN slice cultures

**Figure 2:**
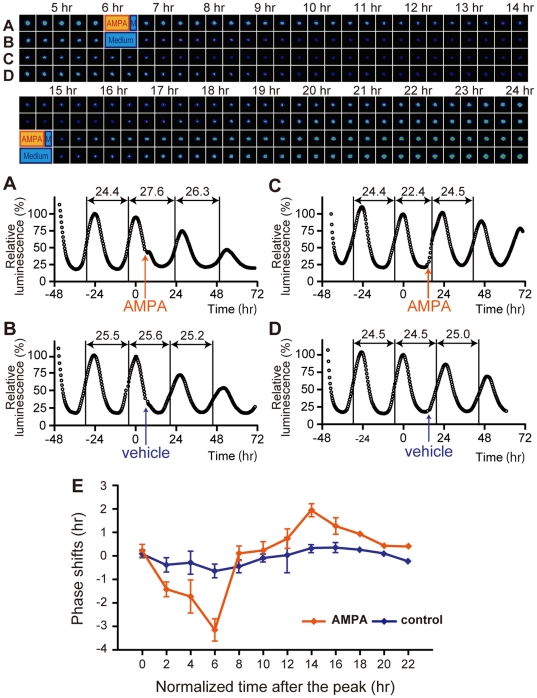
AMPA-induced phase shifts of luminescence rhythms in organotypic SCN slice cultures. Images of the representative results are shown in the upper panels. The corresponding graphs are shown below, defining the second peak values (time 0) as 100%. AMPA application, (**A**) at 6 hr or (**C**) at 14 hr after the peak of the luminescence, induced phase delays and advances, respectively. Control medium treatment without AMPA (**B**) at 6 hr or (**D**) at 14 hr after the peak had no effect on the phase. *p*<0.01 (both at 6 hr and at 14 hr, AMPA vs. control, Student's *t*-test). To calculate the period length, each middle point between peak and trough in the increasing phase was first determined, and the time at the middle point was subtracted by the time at the previous middle point. (**E**) PRC obtained with SCN slice cultures stimulated by AMPA application. The x axis represents the normalized time after the peak (1 normalized hour  =  free running period/24 hr). The y axis represents the magnitude of phase shifts normalized by multiplying each shift in hour by the factor of 24 hr/free-running period. Plus and minus values mean phase advances and delays, respectively. Each value is the Mean ± SEM. The data obtained from multiple SCN slices during two hours were averaged. Hours shown on the x axis represent the middle of each two hours interval. One-way ANOVA revealed significant differences in PRC amplitudes obtained by AMPA application, but not in that obtained by control application (see Materials and Method). Post-hoc analysis using Scheffe's multiple comparisons revealed that the magnitude of AMPA-induced phase shifts at 6 hr was significantly different from the magnitudes at all other time points except at 2 and 4 hr (*p*<0.01).

Next, we examined whether the AMPA-induced behavioral phase-shifts are reproduced in phase-shifts of core clock oscillations, using organotypic SCN slice cultures obtained from *Per1-luc* transgenic mice [14]. When AMPA was applied 6 hr after the peak point of the luminescence (i.e. during the decreasing phase), the next peak was significantly phase delayed compared to control medium application (AMPA, −3.18±0.45 hr, n = 7; control, −0.68±0.26 hr, n = 3) **(** **Figure 2A, B** **)**. In contrast, when AMPA was applied 14 hr after the peak (i.e. between the trough and the next increasing phase), the next peak was significantly phase advanced (AMPA, +1.92±0.24 hr, n = 3; control, +0.31±0.14 hr, n = 3) **(** **Figure 2C, D** **)**.

Since we found phase-dependent phase shifts of core clock oscillations using this *in vitro* real-time monitoring system, we systemically analyzed the AMPA effects over the 24 hours. **Figure 2E** shows AMPA-induced phase-shifts at various time points. The directions and magnitude of AMPA-induced phase shifts were dependent on the circadian phase. AMPA applications at 2–6 hr after the peak caused phase delays, whereas the applications at 14–16 hr caused phase advances. The contour of the phase-response curve (PRC) corresponds well with the PRC obtained with light-induced phase shifts of locomotor activity rhythms *in vivo* [24] or with NMDA-induced phase shifts in cultured SCN slices [14].

## Discussion

AMPAR activation is believed to be just a prerequisite for NMDAR activation which eventually leads to neuronal firings and physiological changes such as long-term potentiation formation in hippocampal neurons [17]. In this study, however, we showed that AMPA microinjection into the SCN *per se* resulted in phase delays of locomotor activity rhythms and phase-shifts in core clock gene oscillations in organotypic SCN slice cultures. Still, since a NMDAR antagonist inhibited the AMPA-induced phase-shift, the following activation of NMDAR must be necessary for the AMPA-induced phase-shift. Taken together with the previous observations that NMDA-induced behavioral phase shifts are also attenuated by AMPAR antagonists [11], [28], we propose that AMPAR and NMDAR signaling reciprocally regulate glutamatergic signaling and determine the magnitude of phase-shifts of behavioral rhythms.

AMPA-induced phase-dependent phase-shifts were observed both in *in vivo* behavioral rhythms and in *in vitro* core clock oscillation rhythms. However, some differences were observed: large phase-delays caused by AMPA in the early subjective night and no phase-shift in the subjective day are commonly observed, but the phase-advances during the late subjective night were only observed *in vitro*. Although several technical issues, such as the concentration of agonist used and method of injection, may explain these discrepancies, it is likewise possible that the presence of inhibitory afferents to the SCN may cause the differences observed between our *in vitro* and *in vivo* experiments. Actually, Mintz and coworkers reported that the degree of NMDA-induced phase-advances during the late subjective night was lower than that of light pulse-induced, although the phase-delays during the early subjective night obtained by both methods were quite similar [11]. Interestingly, Moriya and coworkers reported that aniracetam, an enhancer of AMPAR activity, augmented light-induced phase-shifts at CT14, but not at CT20 [21]. These findings strongly suggest that light-induced phase-advances during the late subjective night need not only glutamatergic stimuli, but also additional factor(s).

In this point, it is noteworthy that abundant inhibitory serotonergic inputs from the midbrain innervate the retinal terminals and retinorecipient cells in the SCN [29]. Since serotonergic inhibitory signals are regulated in a circadian manner and are highest in the late subjective night in the SCN [30], AMPAR activation *in vivo* was not sufficient to induce phase-advances during the late subjective night. However, in the SCN slice cultures where inhibitory serotonergic afferents were mechanistically eliminated, AMPA application might be capable of inducing phase-advances. Besides, the effect of SCN-rich astrocytes *in vivo* [31] and *in vitro* [32] might have some effects on the discrepancy between *in vivo* and *in vitro,* since astrocytes regulate glutamate signaling through glutamate uptake from and/or glutamate release to synapses [33].

In this study, we observed AMPA-induced phase-dependent phase-shifts both in locomotor behavioral rhythms and in core-clock transcription oscillations in SCN slice cultures. These data suggest that the activation of AMPAR is a critical step in behavioral entrainment to light-dark cycles, and highlight the contributions of AMPAR in glutamatergic signaling, which have been underestimated behind NMDAR signaling.

## Materials and Methods

### Animals and Monitoring Behavioral Rhythms

Male C57BL/6 mice at 7–8 weeks age (JAPS, Osaka, Japan) were acclimated for at least one week in an environment with a 12-hr light and 12-hr dark cycle, maintained at 22±2°C, with food and water provided *ad libitum*. Locomotor activity was detected by passive infrared sensors (FA-05 F5B; Omron, Kyoto, Japan). Data were collected and analyzed with Chronobiology kit (Stanford Software Systems, Stanford, CA), as described previously [34]. Circadian time and phase shifts of activity rhythms were analyzed with Clocklab software (Actimetrics, Wilmette, IL). All animal procedures described in this study were approved by the Animal Research Committee of Kyoto University (2010-43) and The Committee for Animal Research of Kobe University (P060601).

### *In Vivo* AMPA and/or Antagonist Microinjection

Mice were deeply anesthetized with a cocktail of ketamine (50 mg/kg) and xylazine (20 mg/kg) and a small hole was drilled 0.4 mm caudal from bregma. A 5.0 mm length guide cannula was stereotaxically implanted, aimed at the SCN, and a dummy cannula was inserted into the guide cannula until AMPA and/or antagonist microinjection. After guide cannula implantation, mice were housed in DD to establish stable free-running locomotor activity rhythms. After 10 days of stable behavioral rhythms, mice were briefly anesthetized with ether, and microinjected using a 5.5 mm length injection needle connected to a 10 µl Hamilton syringe via a polyethylene tube, in dim red light illumination environment. 1 µl of 0.25 mM AMPA (Tocris, Ellisville, MO), 0.5 mM NBQX (Tocris), 0.5 mM AP5 (Tocris), 0.25 mM AMPA plus 0.5 mM NBQX, 0.25 mM AMPA plus 0.5 mM AP5, or artificial cerebrospinal fluid as vehicle (147 mM NaCl, 4 mM KCl, 1.2 mM CaCl_2_, pH 7.0) was injected at a rate of 0.2 µl/min at CT14 or CT22. AMPA or antagonist was dissolved in artificial cerebrospinal fluid. AMPA plus either NBQX or AP5 were simultaneously microinjected as a drug cocktail. After each injection, the needle was left in place for at least 2 min.

### *In Situ* Hybridization

The induction of *Per1* mRNA was measured using a radiolabeled antisense riboprobe covering nucleotides 844–1626 of *Per1* mRNA (Genbank, NM_011065). The corresponding cDNA fragment was cloned and used as a template for riboprobe synthesis. The riboprobes were radiolabeled with [^33^P]UTP (PerkinElmer, Waltham, MA) using a standard protocol for cRNA synthesis. AMPA microinjection was performed at CT14 and mice were again housed in DD until CT15 when mice were sacrificed. *In situ* hybridization was performed according to the method detailed previously [27]. Autoradiography films (Kodak Biomax) were then exposed to the air-dried sections, and signals were quantified by MCID image analyzing system (Imaging Research Inc., Canada) after conversion into the relative optical densities using ^14^C-autoradiographic microscales (Amersham, UK).

For visualizing AMPAR subtypes at the cellular levels, we generated gene-specific probes as follows: *GluR1* antisense probe covering nucleotides 434–1090 of *GluR1* mRNA (Genbank, NM_001113325.1); nucleotides 631–1394 (NM_001039195.1) for *GluR2*; nucleotides 766–1524 (NM_016886.3) for *GluR3* and nucleotides 295–884 (NM_019691.4) for *GluR4*. Digoxigenin-labeled antisense cRNA probes were synthesized using digoxigenin-UTP (Roche Diagnostics GmbH, Mannheim, Germany) following a standard protocol of cRNA synthesis. The sections hybridized with digoxigenin-labeled probes were processed for immunochemistry with the nucleic acid detection kit (Roche Diagnostics GmbH). Signals were visualized in a solution containing nitroblue tetrazolium salt (0.34 mg/ml, Roche Diagnostics GmbH) and 5-bromo-4-chloro-3-indolyl phosphate toluidinium salt (0.18 mg/ml, Roche Diagnostics GmbH).

### *In Vitro* AMPA Application and Bioluminescence Recording

The organotypic SCN slice cultures of *Per1-luc* neonatal transgenic mice (4- to 7-day-old) were obtained as described previously [14]. SCN slice cultures were maintained in a sealed 24-well cell culture plate, with 240 µl of culture medium containing 1 mM luciferin per well at 35°C during bioluminescence recording. For AMPAR stimulation, the SCN slice cultures were transferred at various time points to control medium (50% minimum essential medium, 50% Hank's balanced salt solution, 36 mM glucose, and penicillin/streptomycin), with or without AMPA (5 µM), for 30 min at 35°C, and were then washed three times with control medium for 10 min at 35°C. After the washes, the SCN slice cultures were returned to the original culture medium. Sample sizes at each time point were as below: at 0 hr, control n = 3, AMPA n = 4; 2 hr, 3, 3; 4 hr, 5, 5; 6 hr, 3, 7; 8 hr, 7, 6; 10 hr, 5, 5; 12 hr, 3, 5; 14 hr, 3, 3; 16 hr, 5, 5; 18 hr, 2, 2; 20 hr, 2, 1 and 22 hr, 1, 2, respectively. One-way ANOVA was performed with the data obtained during 0–16 hr time points.

## Supporting Information

Figure S1 The effect of AMPA microinjection at CT6 and CT22 on mouse circadian locomotor activity rhythms. Representative double-plotted actograms of circadian locomotor activity rhythms in mice injected with either vehicle or AMPA. Mice were maintained in constant darkness and microinjections were given (A) at CT6 or (B) at CT22 (marked by asterisks) under dim red light illumination. The magnitude of the phase shifts was calculated as described in the legend of Figure 1. (C) Mean ± SEM of phase shifts induced by AMPA microinjection at CT6 or at CT22. Phase shifts at CT14 are also shown for comparison. Negative and positive values represent phase delays and advances, respectively. p = 0.43 (at CT6) or 0.089 (at CT22) (Student's t-test).
(0.25 MB TIF)
