# Supplementary figures and images for: Activation of AMPA Receptors in the Suprachiasmatic Nucleus Phase-Shifts the Mouse Circadian Clock In Vivo and In Vitro
Source: PLoS One. 2010 Jun 3;5(6):e10951. doi: 10.1371/journal.pone.0010951 (PMC2880614; doi:10.1371/journal.pone.0010951)

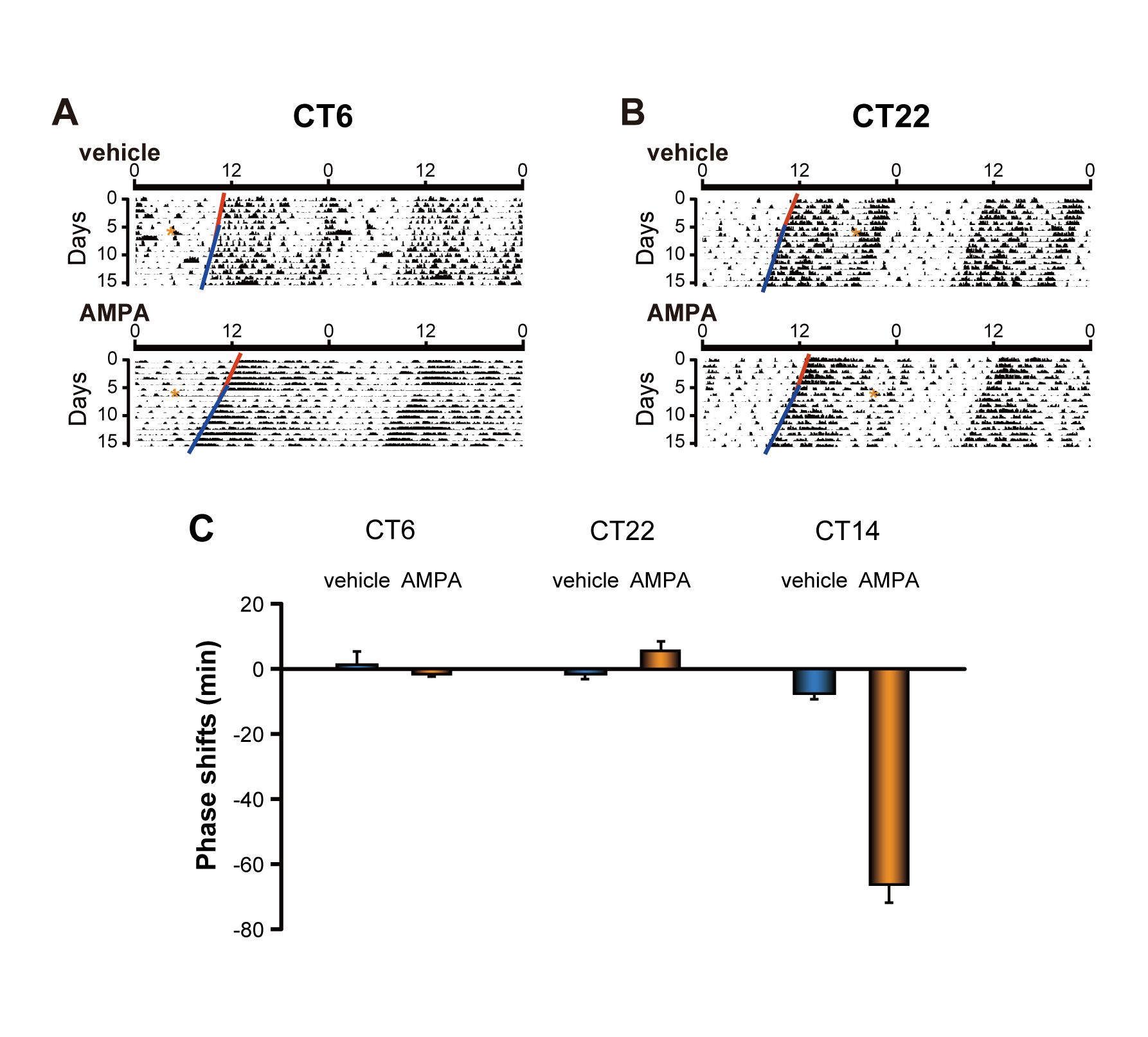

Supplement: Figure S1 — The effect of AMPA microinjection at CT6 and CT22 on mouse circadian locomotor activity rhythms. Representative double-plotted actograms of circadian locomotor activity rhythms in mice injected with either vehicle or AMPA. Mice were maintained in constant darkness and microinjections were given (A) at CT6 or (B) at CT22 (marked by asterisks) under dim red light illumination. The magnitude of the phase shifts was calculated as described in the legend of Figure 1. (C) Mean ± SEM of phase shifts induced by AMPA microinjection at CT6 or at CT22. Phase shifts at CT14 are also shown for comparison. Negative and positive values represent phase delays and advances, respectively. p = 0.43 (at CT6) or 0.089 (at CT22) (Student's t-test). (0.25 MB TIF) [file pone.0010951.s001.tif]
